# Loss of peroxisomal membrane proteins PEX13 and PEX14 disrupts fatty acid oxidation and drives lipid imbalance

**DOI:** 10.1042/BSR20260048

**Published:** 2026-05-22

**Authors:** Navia Vinoy, Gary Huang, Daniel Wallace, V. Nathan Subramaniam

**Affiliations:** 1 Hepatogenomics Research Group, Queensland University of Technology (QUT), QLD 4059, Australia; 2 Centre for Genomics and Personalised Health, School of Biomedical Sciences, Queensland University of Technology (QUT), QLD 4059, Australia; 3 Metallogenomics Laboratory, Queensland University of Technology (QUT), QLD 4059, Australia

**Keywords:** gene silencing, hepatocytes, metabolic dysfunction-associated steatotic liver disease, non alcoholic fatty liver disease, peroxisomes, small interfering RNA

## Abstract

Peroxisomal disorders arise from severe peroxisome dysfunction and are frequently linked to liver pathology. Metabolic dysfunction-associated steatotic liver disease, which affects up to 38% of adults worldwide, has driven extensive efforts to identify genes that contribute to its development. In the present study, we investigated the role of two peroxisomal membrane proteins, PEX13 and PEX14, by performing single and dual small interfering RNA-mediated knockdowns in a liver cell line, HUH-7. Steatosis was induced using free fatty acids, and changes in lipid-metabolism gene expression were assessed by quantitative real-time polymerase chain reaction. Knockdown efficiency reached 90% for both genes, and Oil-Red-O staining confirmed successful induction of steatosis. Both single and combined knockdown of *PEX13* and *PEX14* altered the expression of genes involved in lipid sensing, fatty acid uptake, synthesis, and oxidation. These findings suggest that peroxisomal dysfunction disrupts hepatic metabolic pathways, promoting increased fatty acid uptake and synthesis. Such alterations may contribute to the liver dysfunction observed in patients with peroxisome biogenesis disorders, highlighting the importance of peroxisomal integrity in maintaining lipid homeostasis.

## Introduction

PEX13 and PEX14 are critical peroxins that mediate the import of peroxisomal matrix proteins into peroxisomes, including enzymes integral to fatty acid synthesis, oxidation, and reactive oxygen detoxification [1]. PEX13 and PEX14 form a docking complex on the peroxisomal membrane that serves as a binding site for PEX5, the cargo-loaded receptor protein that transports cargo proteins into peroxisomes [2]. Mutations affecting PEX13 or PEX14 proteins are linked to peroxisome biogenesis disorders, including Zellweger spectrum disorders [3], indicating that these proteins have essential roles in maintaining peroxisomal structure and function.

Disruption of PEX13 results in the accumulation of ubiquitinated PEX5 on peroxisomes, enhanced reactive oxygen species production, and significantly interferes with peroxisomal fatty acid oxidation and plasmalogen biosynthesis [4–6]. Depletion of PEX14 has been shown to result in compromised peroxisomal biogenesis, suppressed insulin secretion, and impaired lipid metabolism and storage, leading to elevated reactive oxygen levels, enhanced lipid peroxidation, and the activation of regulated cell death, including autophagy, apoptosis, and ferroptosis [7,8]. These findings suggest that mutations in these *PEX* genes could disrupt the transport of numerous proteins, including enzymes involved in the synthesis and oxidation of fatty acids.

Hepatic steatosis is characterized by increased fatty acid synthesis and a potential concurrent decrease in fatty acid oxidation, leading to fat accumulation [9,10]. Hepatic steatosis can result in organelle dysfunction, oxidative stress, and apoptosis, thereby contributing to the progression of metabolic dysfunction-associated steatotic liver disease (MASLD) towards metabolic dysfunction-associated steatohepatitis (MASH) and chronic liver disease [11]. Given the essential role of *PEX13* and *PEX14* in peroxisomal fatty acid metabolism, we hypothesized that their deficiency or dysregulation may disrupt fatty acid oxidation and promote lipid accumulation, potentially leading to steatosis and MASLD. In the present study, we investigated the role of peroxisomal genes *PEX13* and *PEX14* in the development of steatosis using the HUH-7 liver cell line.

## Materials and methods

### Cell culture

HUH-7 cells, an immortalized human hepatocellular carcinoma cell line derived from a 57-year-old Japanese male, are widely used as a primary hepatocyte substitute [12]. HUH-7 cells (#JCRB0403; Japanese Cancer Research Resources Bank Cell Bank; CellBank Australia) were cultured at 37°C in a 5% CO_2_ humidified incubator (#SCO6AD-2; LabGear, South Melbourne, Victoria, Australia) and were maintained in Roswell Park Memorial Institute 1640 medium (#11875093; Gibco) supplemented with 10% FBS (#A5669701; Gibco).

### siRNA knockdown studies

Small interfering RNA (siRNA) for the peroxisomal genes *PEX13* and *PEX14* (Table 1) was purchased from Integrated DNA Technologies (IDT) (Chatswood, New South Wales, Australia). The cells were transfected for 48 h with a single siRNA or both siRNAs to simulate single or double gene knockdown [13]. The gene knockdown experiments were conducted using Lipofectamine RNAiMAX (#13778150; Invitrogen; Thermo Scientific), as per the manufacturer’s protocol, with 10 pmol of siRNAs targeting *PEX13* or *PEX14* (Table 1).

**Table 1:** siRNA sequences

Gene	Sequence (5′ → 3′)
*PEX 13*	Sense: CGGUGGAAUCAAGUAAAGUUUCCAA
	Antisense: UUGGAAACUUUACUUGAUUCCACCGUU
*PEX14*	Sense: GUCCCAGAAUAUCAACGAACUCAAG
	Antisense: CUUGAGUUCGUUGAUAUUCUGGGACUC
Nonspecific control	Sense: UUCUCCGAACGUGUCACGUdTdT
	Antisense: ACGUGACACGUUCGGAGAAdTdT

### Induction of steatosis

A MASLD-like environment was promoted by treating the cells for 24 h with free fatty acids (FFAs) composed of oleate (#O7501; Sigma–Aldrich) and palmitate (#P9767; Sigma–Aldrich) in a 2:1 ratio. A 12 mM FFA stock solution was prepared with 8% fatty acid-free bovine serum albumin (#A8806; Sigma–Aldrich), which was subsequently diluted with culture media to the desired working concentration (1 mM) [14–17].

### Oil Red O staining

Cells were washed twice with 1× phosphate-buffered saline (PBS) (#09-2051-100; Astral, Gymea, New South Wales, Australia), and then fixed for 15 min at room temperature (RT) with 3% paraformaldehyde (PFA) (#P6148; Sigma–Aldrich), which was diluted in PBSCM (PBS with 1 mM calcium chloride (#C3881; Sigma–Aldrich) and 1 mM magnesium chloride (#AJA296-500G; AJAX FineChem; Thermo Scientific). The fixed cells were then washed twice with deionized water, followed by incubation with 60% isopropanol (#AJA425-2.5LPL; AJAX FineChem) for 5 min at RT. The samples were then incubated in Oil Red O (#O0625; Sigma–Aldrich) staining solution for 15 min at RT. The Eclipse Ts2 inverted microscope (Nikon, Melville, New York, U.S.A.) was used to image the cells.

### Cell metabolic activity assay

The 3-(4,5-dimethylthiazol-2-yl)-2,5-diphenyltetrazolium bromide (MTT) assay was used to assess the metabolic activity of cells following peroxisomal gene knockdown and FFA treatment. An MTT stock solution was prepared by dissolving 5 mg of MTT powder (#M5655-100MG; Sigma Aldrich) in 1 ml of PBS and diluting with 10 ml of Opti-MEM. The cells were treated with the MTT solution for 3–4 h, allowing for crystal formation, and 200 μl of 100% dimethyl sulfoxide was added to stop the reaction. Quantification was performed using a CLARIOstar microplate reader, measuring absorbance at 540 and 690 nm.

### Immunofluorescence staining and flow cytometry

Cells were fixed with 3% PFA for 15 min at RT, quenched with NH_4_Cl (ammonium chloride; #A4514; Sigma–Aldrich) to free any aldehyde groups, and then permeabilized with 0.1% saponin (#558255; Calbiochem) for 15 min at RT. Subsequently, cells were incubated with primary PEX13 and PEX14 antibodies (both gifts from Denis Crane, Griffith University, made in rabbit), diluted 1:1000 and 1:2000, respectively, with fluorescence dilution buffer (FDB) (5% donkey serum; #566460; Merck, Bayswater, VIC, Australia), 5% FBS, and 2% bovine serum albumin (#A7906; Sigma–Aldrich) in PBSCM for 60 min at RT. The cells were then incubated with secondary donkey anti-rabbit 488 (#A21206; Invitrogen; Thermo Scientific) diluted 1:100 in FDB for another 60 min at RT. Subsequently, the cells were resuspended with FACS buffer (2% FBS in PBS) and then analyzed on the FACS Celesta. The fluorescent data were analyzed using FlowJo software (BD Biosciences).

### RNA extraction, cDNA synthesis, and qRT-PCR

Total ribonucleic acid (RNA) was isolated from cells using TRIzol (#15596018; Invitrogen; Thermo Scientific), as per the manufacturer’s protocol. The SensiFAST cDNA synthesis kit (#BIO-65054; Bioline) or the High-Capacity cDNA Reverse Transcription Kit (#4368814; Applied Biosystems) was used to perform first-strand cDNA synthesis, as per the manufacturer’s protocol. Quantitative real-time polymerase chain reaction (qRT-PCR) was performed using the QuantiNova SYBR Green PCR kit (#208057; QIAGEN, Chadstone, VIC, Australia), as per the manufacturer’s protocol, on the QuantStudio 7 Pro Real-Time PCR system (#A43183; Applied Biosystems; Thermo Scientific). All primers were purchased from IDT (Chatswood, New South Wales, Australia). The primer sequences are detailed in Table 2. Gene expression was normalized to the geometric mean of β-actin (*ACTB*) and hypoxanthine-guanine phosphoribosyltransferase (*HPRT*) housekeeper genes. The analysis was performed using the ΔΔCt method.

**Table 2:** Human qRT-PCR primer sequences

Gene	Primer	Sequence (5′ → 3′)
*ACTB*	F	CAGGCACCAGGGCGTG
	R	GCCCACATAGGAATCCTTCTGA
*HPRT*	F	GAAAGGGTGTTTATTCCTCAT
	R	CCCATCTCCTTCATCACAT
*PEX13*	F	CCATGTAGTTGCCAGAGCAG
	R	CATCAAGGCTAGCCAGAAGC
*PEX14*	F	GCCACCACATCAACCAACTG
	R	GTCTCCGATTCAAAAGAAGTCCT
*CPT1A*	F	TCCAGTTGGCTTATCGTGGTG
	R	TCCAGAGTCCGATTGATTTTTGC
*FASN*	F	AAGGACCTGTCTAGGTTTGATGC
	R	TGGCTTCATAGGTGACTTCCA
*ACOX1*	F	CTTCAACCCGGAGCTGCTTA
	R	ATGTTCTCGATCTCTCGGCG
*CD36*	F	CAGGTCAACCTATTGGTCAAGCC
	R	GCCTTCTCATCACCAATGGTCC
*PPARA*	F1	TCACCACAGTAGCTTGGAGC
	R1	GGAACTCTTCAGATAACGGGCT
	F2	ATGGTGGACACGGAAAGCC
	R2	CGATGGATTGCGAAATCTCTTGG
*PPARG*	F	TCGAGGACACCGGAGAGG
	R	CACGGAGCTGATCCCAAAGT
*SREBF1*	F1	CATGGACGAGCCACCCTTC
	R1	GCCGACTTCACCTTCGATGT
	F2	CGGAACCATCTTGGCAACAGT
	R2	CGCTTCTCAATGGCGTTGT

Abbreviations: F, forward; R, reverse; ACTB, β-actin; HPRT, hypoxanthine-guanine phosphoribosyltransferase; PEX 13, peroxisomal biogenesis factor 13; PEX 14, peroxisomal biogenesis factor 14; CPT1A, carnitine palmitoyltransferase 1A; FASN, fatty acid synthase; ACOX1, acyl-CoA oxidase 1; CD36, cluster of differentiation 36; PPARA, peroxisome proliferator activated receptor α; PPARG, peroxisome proliferator activated receptor γ; SREBF1, sterol regulatory element binding transcription factor 1.

### Statistical analysis

Statistical analysis was performed by using GraphPad Prism software (version 6.0-10.0; San Diego, U.S.A.). Statistical analysis between different groups and controls was performed using one- or two-way analysis of variance (ANOVA). Post-hoc analysis was conducted on multivariate analyses using Tukey corrections. *P*-values <0.05 were considered statistically significant (*P <0.05; **P <0.01; ***P <0.001; ****P <0.0001). Due to batch effects between* in vitro* biological replicates, the data in each biological replicate were normalized to the control sample for fold-change analysis.

## Results

### siRNA knockdown of peroxisomal genes PEX13 and PEX14

**Figure 1:**
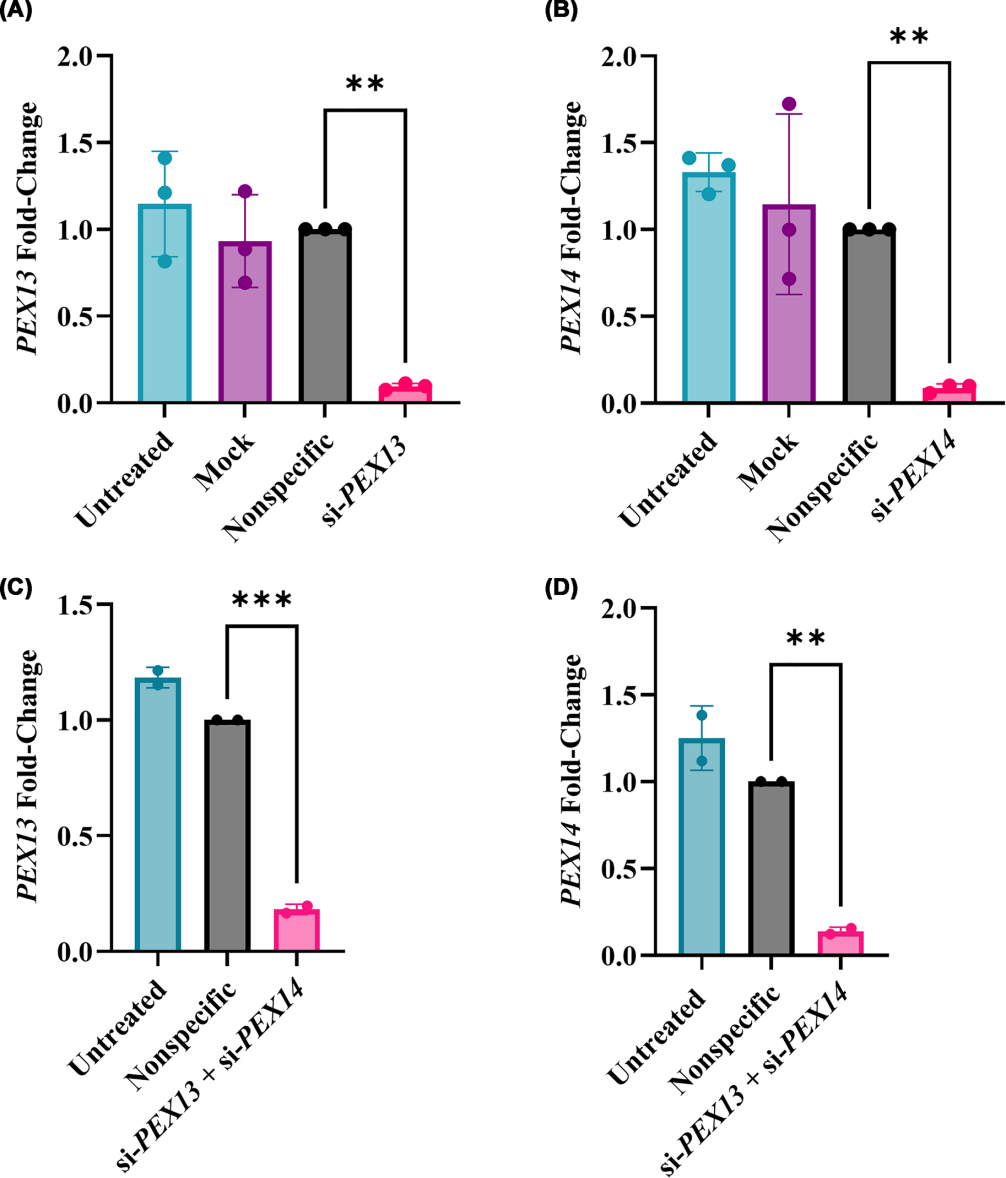
siRNA knockdown of peroxisomal genes *PEX13* and *PEX14* Expression of peroxisomal genes (**A**,** C**) *PEX13* or (**B**, **D**) *PEX14* was quantified by qRT-PCR in HUH-7 cells after 48 h of single si-*PEX13* or si-*PEX14* transfection (A, B) or double knockdowns (C, D), respectively. The mRNA expressions were normalized to the nonspecific siRNA control. Experiments were performed with three biological replicates (*n* = 3) with technical triplicates. One-way ANOVA was conducted with post-hoc Tukey correction. The graphs show the mean and standard error of the mean (SEM). Statistically significant differences were denoted as ** (*P* <0.01) and *** (*P* <0.001).

siRNA-mediated single gene knockdowns resulted in approximately an 80%–90% decrease in *PEX13* or *PEX14* gene expressions compared with the nonspecific control (*P* = 0.001 or *P* = 0.01, respectively) (Figure 1A,B). In double gene knockdown cells, PEX13 or PEX14 gene expression was also about 80%–88% lower than the nonspecific control, with or without FFA treatment (*P* <0.0001 or *P* <0.01, respectively) (Figure 1C,D).

**Figure 2:**
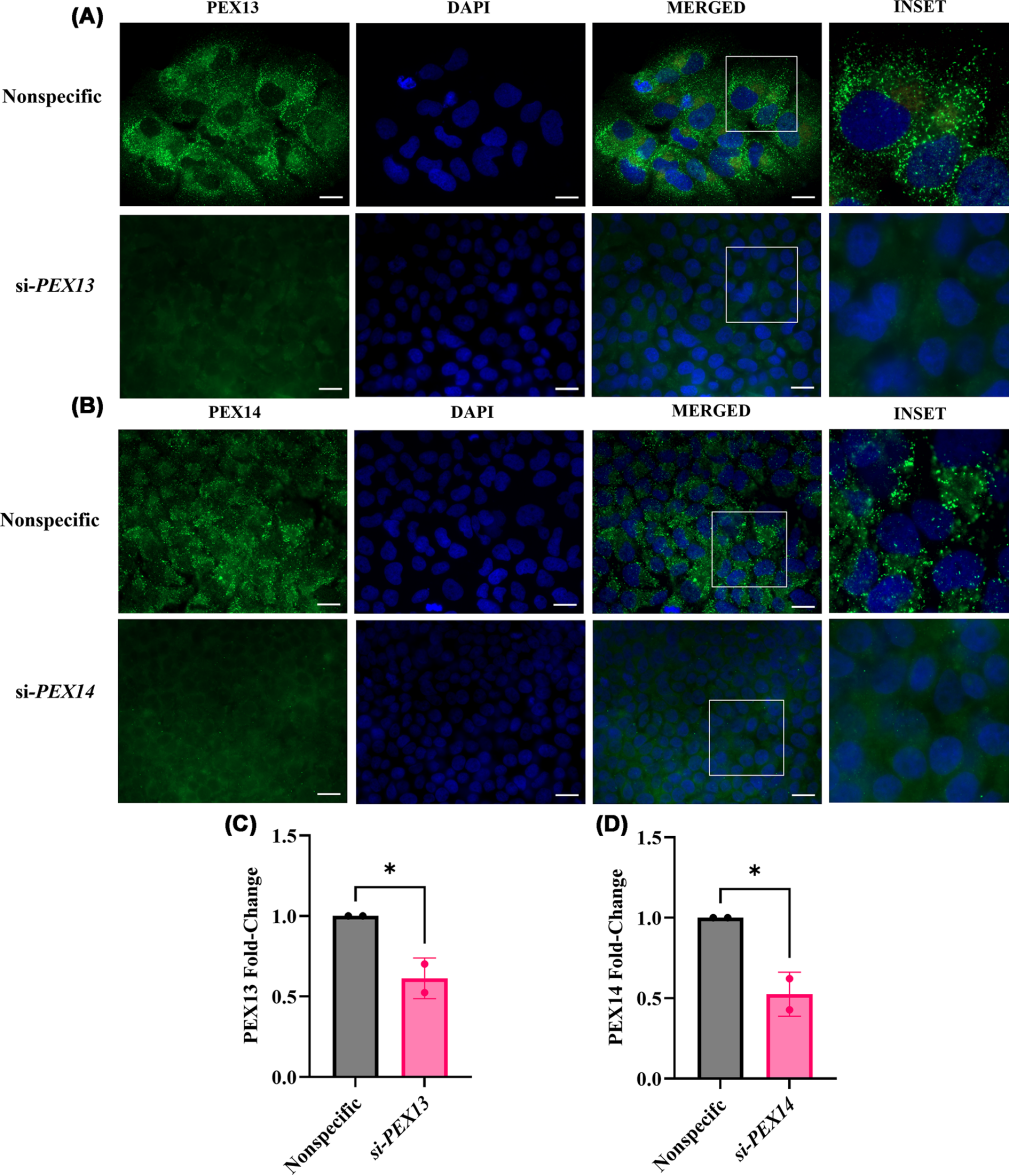
Protein expression of PEX13 and PEX14 in HUH-7 cells Localization and protein expression of (**A**,** C**) PEX13 or (**B**,** D**) PEX14 were assessed by immunofluorescence microscopy or flow cytometry on HUH-7 cells after 48 h of si-*PEX13* or si-*PEX14* transfection, respectively. The image shows immunofluorescent staining for (A) PEX13 or (B) PEX14 protein localization (in green) and nucleus (DAPI; in blue). The inset shows a 135% digital enlargement of the boxed region, displaying the characteristic punctate structures of (A) PEX13 and (B) PEX14. Slides were imaged on the Zeiss Z2 Axioimager microscope using a 63× oil objective. Scale bar is 20 μm. The protein expression of (C) PEX13 or (D) PEX14 was normalized to the nonspecific siRNA control. Experiments were performed with two biological replicates (*n* = 2) with technical triplicates. An unpaired *t*-test was conducted. The graphs show the mean and SEM. Statistically significant differences are denoted as * (*P* <0.05).

PEX13 and PEX14 are localized to cytoplasmic punctate structures, representing peroxisomal membrane sites [4]. A marked decline in punctate structures was observed following si-*PEX13* or si-*PEX14* transfection (Figure 2A,B), suggesting disrupted peroxisomal membrane protein localization. These findings were further supported by flow cytometry, which exhibited a marked reduction in PEX13 and PEX14 protein expression (*P* <0.0001; unpaired *t*-test) (Figure 2C,D).

### Effect of PEX13 and PEX14 knockdown on lipid accumulation

**Figure 3:**
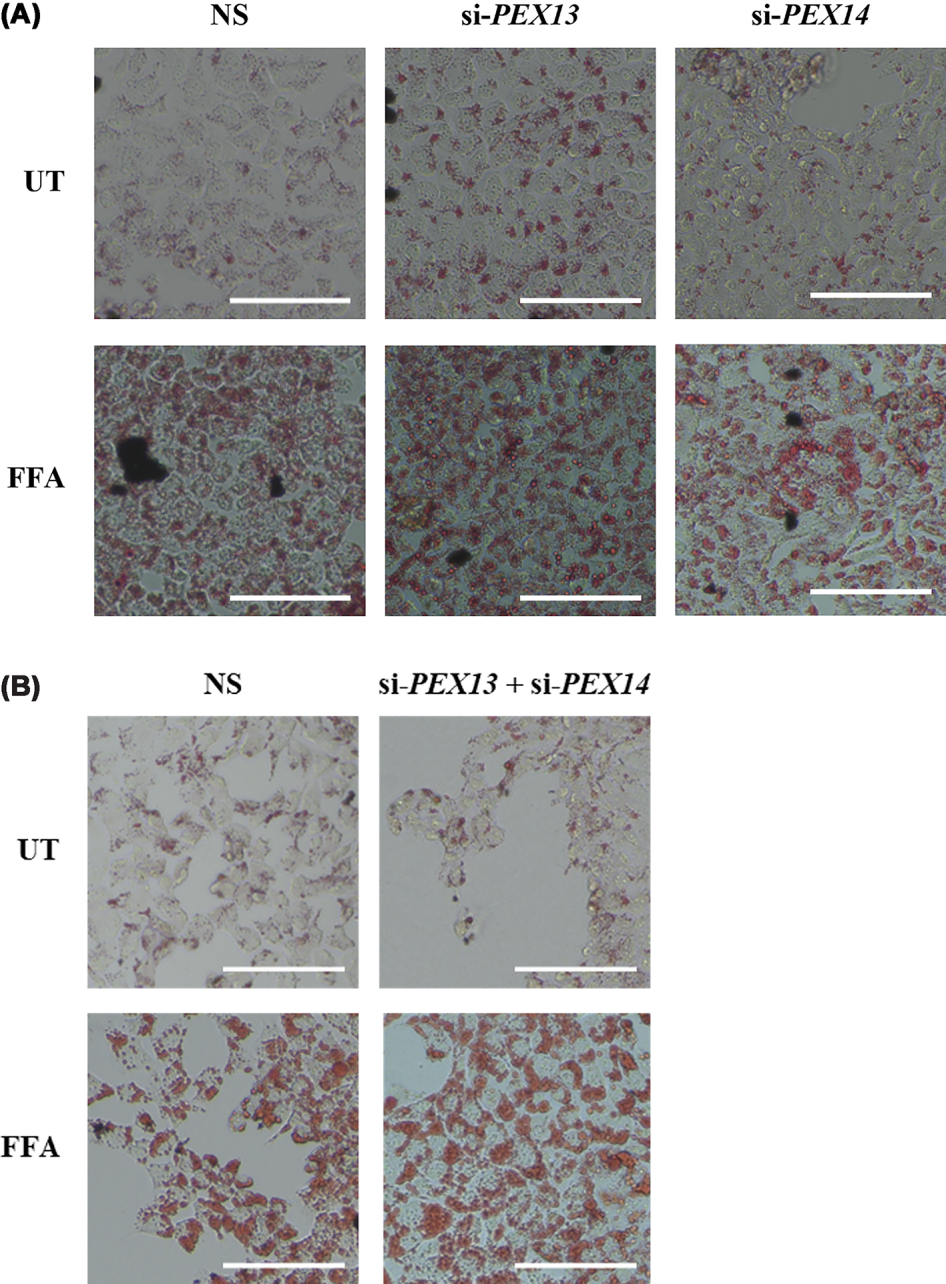
Visualization of lipid accumulation in HUH-7 cells following *PEX* gene knockdown and FFA treatment Oil Red O staining of HUH-7 cells transfected with either a nonspecific (NS) control siRNA, (**A**) si-*PEX13* or si-*PEX14*, or (**B**) a combination of si-*PEX13* and si-*PEX14* for 48 h and treated with or without 1 mM FFA for 24 h. Images were obtained using a Nikon Eclipse Ts2 inverted microscope. Representative images are shown (*n* = 3). Scale bar = 250 μm.

To investigate the consequences of depletion of PEX13 and PEX14 in HUH-7 cells on lipid accumulation, cells were treated with PEX13 or PEX14 siRNA followed by a 24-h treatment with 1 mM FFA. This concentration of FFA was chosen as it resulted in maximal lipid accumulation (Supplementary Data Figure S1). Following this, a metabolic activity assay was undertaken to measure the extent of cell death after 48 h of peroxisomal gene knockdown and 24 h of free fatty acid treatment (Supplementary Data Figure S2). Increased lipid accumulation as measured by Oil Red O staining was observed following FFA treatment in all siRNA conditions (Figure 3A,B).

### Effect of PEX13 and PEX14 depletion on lipid metabolism-associated genes

To understand the effects of PEX13 and PEX14 depletion on expression of genes associated with lipid metabolism, qPCR was performed to quantify the expression of carnitine palmitoyltransferase 1A (*CPT1A*) and acyl-CoA oxidase 1 (*ACOX1*), which encode crucial enzymes involved in mitochondrial and peroxisomal β-oxidation [18]. *CPT1A* encodes a rate-limiting enzyme in the liver for long-chain fatty acid β-oxidation [19]. Up-regulation of this gene is associated with increased mitochondrial ROS [20]. The *ACOX1* gene encodes an enzyme that participates in the peroxisomal fatty acid beta-oxidation pathway, which is responsible for the degradation of fat molecules, specifically very long-chain fatty acids [21].

**Figure 4:**
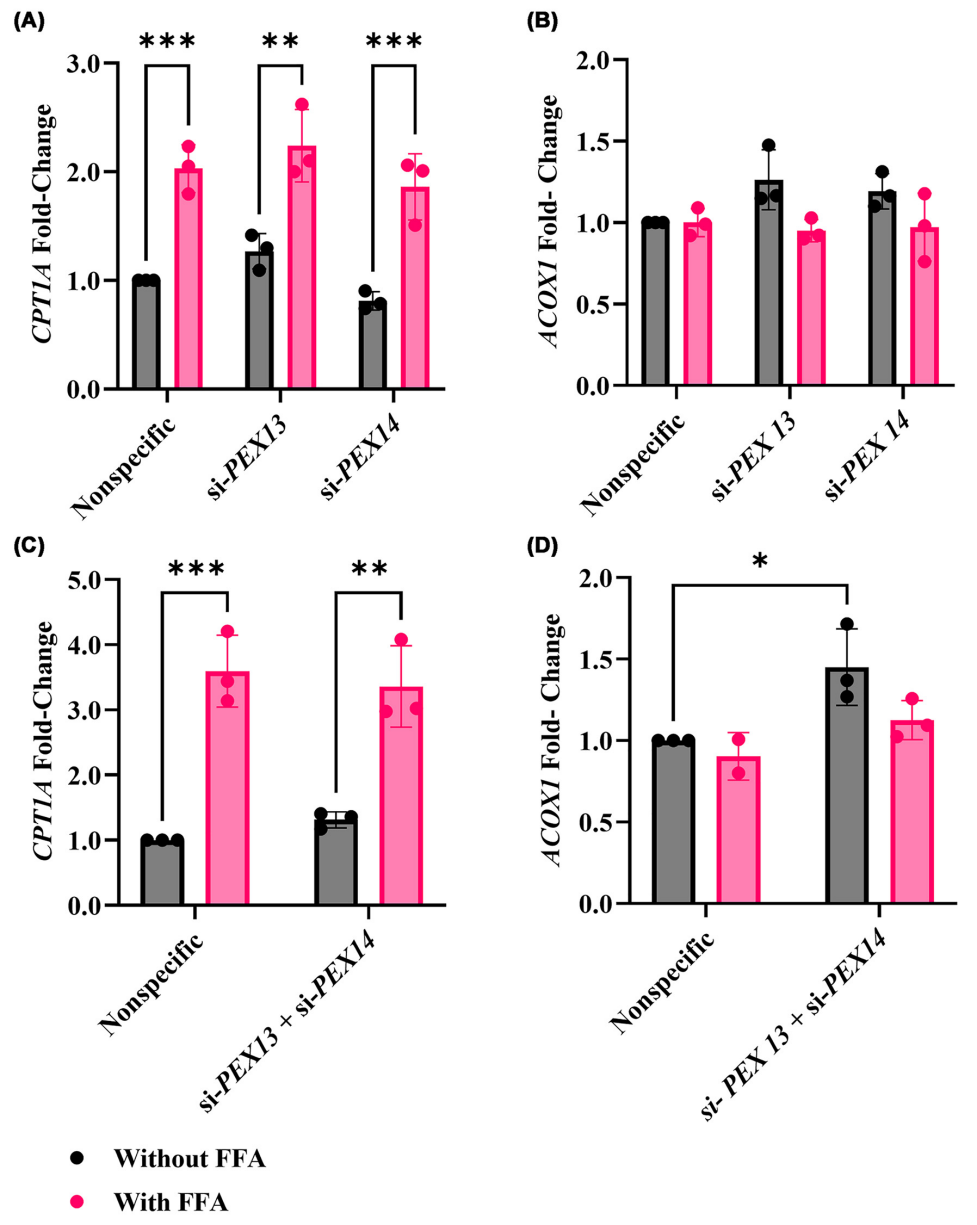
Effect of *PEX13* and *PEX14* knockdown combined with FFA treatment on *CPT1A* and *ACOX1* expression in HUH-7 cells Expression of lipid-oxidation genes was quantified by qRT-PCR in HUH-7 cells after 48 h of si-*PEX13* or si-*PEX14* transfection, or combined knockdown of both genes, with or without 1 mM of FFAs. Panels (**A**) and (**C**) show *CPT1A* expression after single and combined knockdown, respectively, whereas panels (**B**) and (**D**) show *ACOX1* expression under the same conditions. The mRNA expressions were normalized to the nonspecific siRNA control. Experiments were performed with three biological replicates (*n* = 3) with technical triplicates. Two-way ANOVA was conducted with post-hoc Tukey correction. The graphs show the mean and SEM. Statistically significant differences are denoted as * (*P* <0.05), ** (*P* <0.01), and *** (*P* <0.001).

FFA treatment resulted in about a two-fold higher *CPT1A* gene expression (all *P* <0.01) (Figure 4A,C). However, there was no significant difference in *CPT1A* gene expression between si-*PEX13* or si-*PEX14* and the nonspecific control, with or without FFA treatment. There were no significant differences in *ACOX1* gene expression, regardless of condition, in the single gene knockdown (Figure 4B). However, basal *ACOX1* expression was significantly higher after concurrent knockdown of *PEX13* and *PEX14* genes compared with the nonspecific control (*P* = 0.03) (Figure 4D).

We next examined the expression of genes involved in *de novo* lipogenesis (DNL), which plays a crucial role in MASLD; its up-regulation leads to increased synthesis of fatty acids, resulting in liver fat accumulation [22]. Fatty acid synthase (FASN) plays a crucial role in determining the maximum hepatic capacity for the DNL process by catalysing the final step [23]. Sterol regulatory element-binding transcription factor 1 (SREBF1), a member of the transcription factor class known as SREBPs, actively participates in the synthesis of fatty acids, triglycerides, and cholesterol [24]. Peroxisome proliferator-activated receptor gamma (PPARG) is a nuclear receptor that controls lipid metabolism by activating SREBF1, which in turn up-regulates FASN, leading to increased fatty acid synthesis and lipid storage, ultimately leading to steatosis in hepatocytes [25].

**Figure 5:**
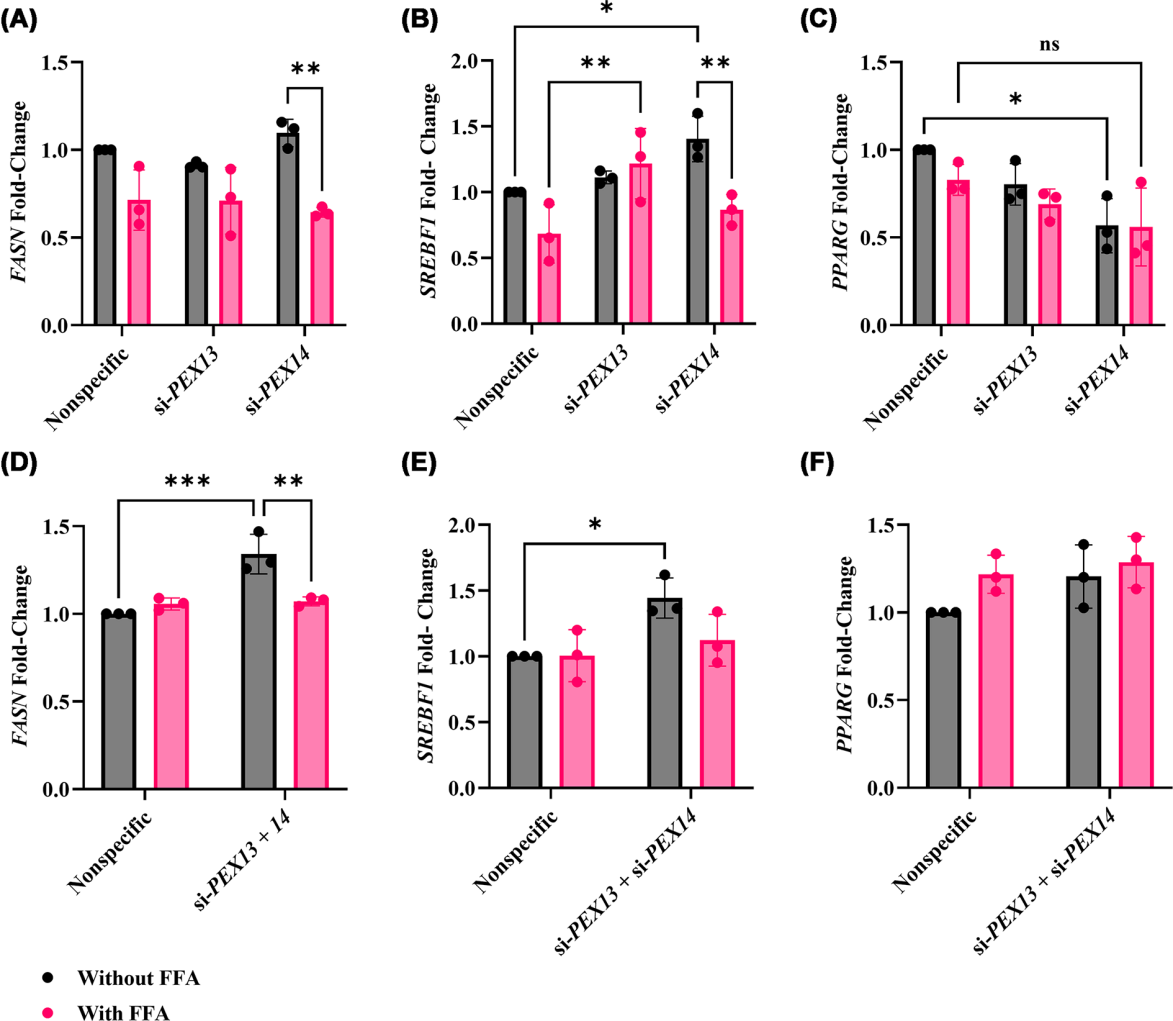
Effect of *PEX13* and *PEX14* knockdown combined with FFA treatment on *FASN*, *SREBF1*, and *PPARG* gene expression in HUH-7 cells Expression of lipid synthesis genes was quantified by qRT-PCR in HUH-7 cells after 48 h of si-*PEX13* or si-*PEX14* transfection, or combined knockdown of both genes, with or without 1 mM FFA. Panels (**A**) and (**D**) show *FASN* expression after single and combined knockdowns, respectively. Panels (**B**) and (**E**) show *SREBF1*, while (**C**) and (**F**) represent *PPARG* expression under the same conditions. The mRNA expressions were normalised to nonspecific siRNA control. within each biological replicate. Experiments were performed with three biological replicate (*n* = 3) with technical triplicates. Two-way ANOVA was conducted with post-hoc Tukey correction. The graphs show the mean and SEM. Statistically significant differences are denoted as * (*P* <0.05), ** (*P* <0.01), *** (*P* <0.001), and ns = not significant.

There were no significant differences in *FASN* expression between si-*PEX13* or si-*PEX14* and the nonspecific control, regardless of FFA treatment (Figure 5A). FFA treatment of si-*PEX14* transfected cells resulted in 41.2% lower *FASN* gene expression compared with untreated si-*PEX14* cells (*P* = 0.003). Concurrent knockdown of *PEX13* and *PEX14* resulted in 1.34-fold higher basal *FASN* gene expression compared with the nonspecific control (*P* = 0.001) (Figure 5D). Although *FASN* gene expression was significantly reduced by 20.1% following FFA treatment in these cells compared with without (*P* = 0.003), this was not significantly different compared with the FFA-treated nonspecific control.

Following FFA treatment, *SREBF1* gene expression was about 1.79-fold higher in si-*PEX13*-transfected cells compared with the FFA-treated nonspecific control (*P* = 0.02) (Figure 5B). *SREBF1* gene expression was significantly increased in si-*PEX14*–transfected cells, showing an approximately 40.4% increase relative to the nonspecific control (*P* = 0.03). Similar to *FASN*, *SREBF1* gene expression was also significantly lower in FFA-treated si-*PEX14*-transfected cells compared with untreated si-*PEX14*-transfected cells (about 38.4%; *P* = 0.02). Concurrent knockdown of *PEX13* and *PEX14* resulted in 1.44-fold higher basal *SREBF1* gene expression compared with the nonspecific control (*P* = 0.04) (Figure 5E).

In untreated cells transfected with si-*PEX14*, *PPARG* gene expression was about 43.2% lower than in the untreated non-specific control (*P* = 0.02) (Figure 5C). *PPARG* gene expression remained suppressed in si-*PEX14* transfected cells following FFA treatment compared with the FFA-treated nonspecific control (about 32%; *P* = 0.19), although this difference did not reach statistical significance. This suggests that *PEX14* knockdown reduces *PPARG* expression and that FFA treatment does not further modify *PPARG* levels beyond the effect of the knockdown. However, there was no significant difference in *PPARG* gene expression between si-*PEX14* transfected cells with or without FFA. *PPARG* gene expression was not significantly impacted by the double gene knockdown (Figure 5F).

We next examined expressions of lipid sensor and lipid uptake genes. Cluster of differentiation 36 (CD36) acts as a receptor for long-chain fatty acids, thereby participating in lipid metabolism [26]. CD36 is responsible for cellular uptake of free fatty acids, contributing to hepatic steatosis, which can subsequently lead to the progression of MASH [27,28]. Peroxisome proliferator-activated receptor alpha (*PPARA*) plays a regulatory role in the advancement of MASLD by controlling the lipogenic pathways of the liver [29,30].

**Figure 6:**
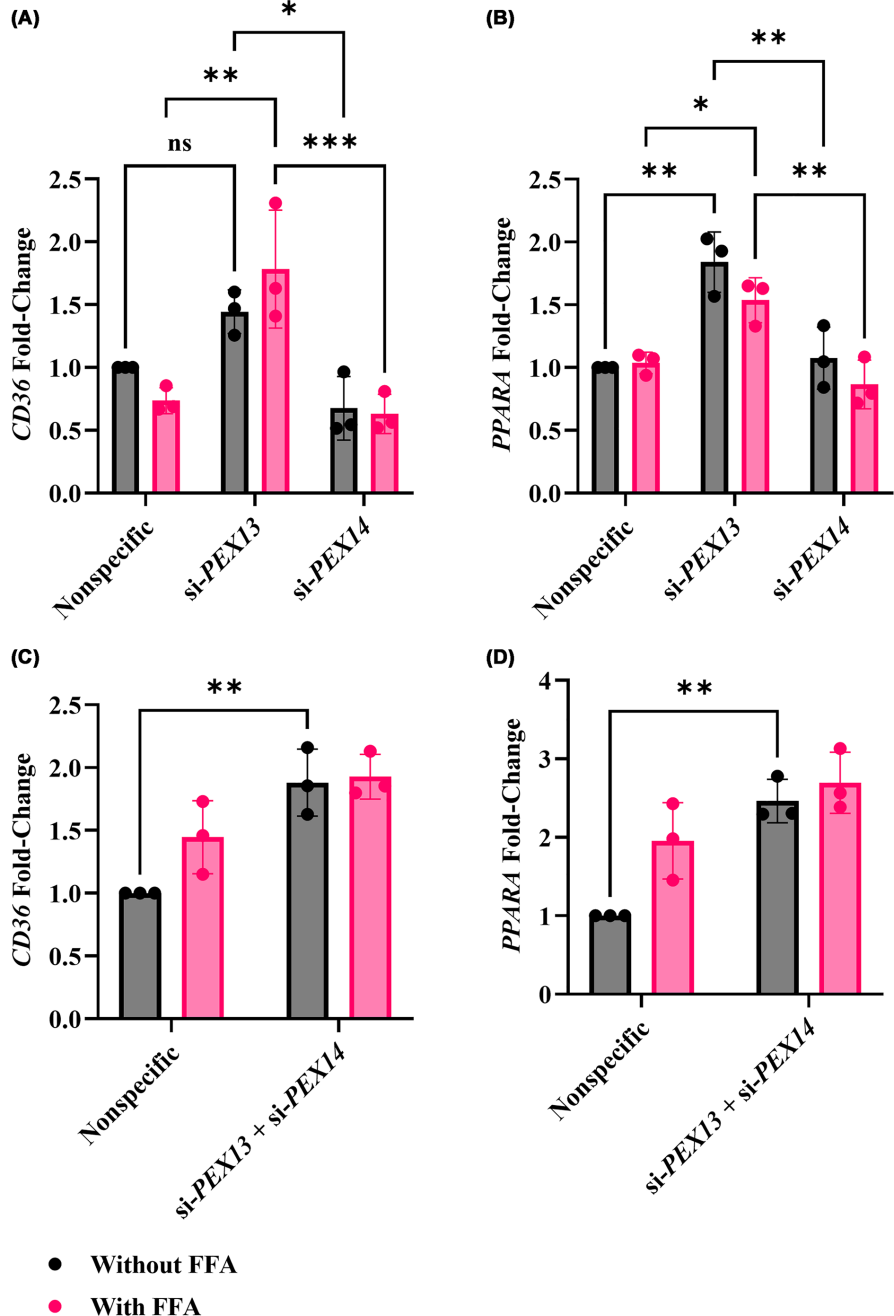
Effect of *PEX13* and *PEX14* knockdown combined with FFA treatment on *CD36* expression in HUH-7 cells Expression of *CD36* and *PPARA* genes was quantified by qRT-PCR in HUH-7 cells after 48 h of si-*PEX13* or si-*PEX14* transfection, or combined knockdown of both genes, with or without 1 mM FFA. Panels (**A**) and (**C**) show *CD36* expression after single and combined knockdown, respectively, whereas panels (**B**) and (**D**) show *PPARA* expression under the same conditions. The mRNA expressions were normalised to nonspecific siRNA control. within each biological replicate. Experiments were performed with three biological replicate (*n* = 3) with technical triplicates. Two-way ANOVA was conducted with post-hoc Tukey correction. The graphs show the mean and SEM. Statistically significant differences are denoted as: * (*P* <0.05), ** (*P* <0.01), *** (*P* <0.001), and ns = not significant.

Although a supplementary unpaired *t*-test indicated *CD36* expression was markedly elevated in si*-PEX13–*transfected cells, showing a 1.44-fold increase compared with the untreated nonspecific control (*P* = 0.01), this change did not reach statistical significance in the two-way ANOVA used for the main analysis. Under FFA-treated conditions, si*-PEX13* cells also exhibited higher *CD36* expression—approximately 2.42-fold relative to the FFA-treated nonspecific control (*P* = 0.002) (Figure 6A). *PPARA* expression was significantly increased in *PEX13-*knockdown cells compared with the nonspecific control, both in the absence and presence of FFA. In untreated cells transfected with si-*PEX13*, *PPARA* levels increased by approximately 1.84-fold (*P* = 0.001), and under FFA-treated conditions, expression remained higher, showing a 1.48-fold increase relative to the corresponding control (*P* = 0.05) (Figure 6B).

There was no significant difference in *CD36* or *PPARA* gene expression between si-*PEX14*-transfected cells and the nonspecific control, with or without FFA treatment (Figure 6A,B). In concurrent knockdown of *PEX13* and *PEX14*, basal *CD36* and *PPARA* gene expressions were significantly higher than the untreated nonspecific control (about 1.88-fold or 2.46-fold; *P* = 0.01 or *P* = 0.003, respectively) (Figure 6C,D).

## Discussion

The present study aims to examine the roles of the peroxisomal genes *PEX13* and *PEX14* in modulating lipid metabolism under lipid-loading conditions in HUH-7 cells. A key objective of the present study was to establish how reduced expression of *PEX13* and *PEX14* affects lipid accumulation, metabolic activity, and the expression of crucial genes involved in fatty acid uptake, synthesis, and oxidation. The purpose of this research was to delineate the unique contributions of *PEX13* and *PEX14* to peroxisomal function and lipid homeostasis by incorporating molecular, cellular, and functional assays.

To determine whether any potential synthetic lethality, genetic buffering, or functional redundancy exists between *PEX13* and *PEX14*, we performed a double knockdown of these genes, along with a single knockdown. By concurrently targeting *PEX13* and *PEX14*, our goal was to disrupt peroxisomal protein import more profoundly than single knockdowns, displaying their synergistic effect in maintaining peroxisomal integrity. The single and concurrent siRNA-mediated silencing of *PEX13* and *PEX14* resulted in a significant decrease in gene expression (approximately 90%), creating a robust model to mimic peroxisomal dysfunction [31]. The observed changes in peroxisomal morphology, especially the decrease in staining following *PEX13* and *PEX14* knockdown, display structural disruptions [32]. This structural modification was supported by a significant reduction in protein expression levels, validated by flow cytometry analysis [4]. Overall, these results suggest that silencing *PEX13* and *PEX14* affects peroxisomal architecture at both the morphological and protein levels, reinforcing their crucial roles in the maintenance and function of peroxisomes.

Following FFA treatment, increased lipid accumulation was observed in HUH-7 cells, with Oil Red O staining plateauing at 1 mM. The present study aligns with prior findings in HepG2 cells and substantiates the hypothesis that hepatocyte lipid storage capacity reaches its maximal effect at this concentration, thereby validating the use of 1 mM FFA for subsequent experimental procedures [33].

The differential expression profiles of lipid metabolism-related genes revealed the unique regulatory roles of *PEX13* and *PEX14*. *CPT1A*, a key mitochondrial β-oxidation gene, was increased following FFA treatment, suggesting a compensatory mechanism to lipid accumulation [30]. This observation aligns with previous findings in liver injury research. The up-regulation of *ACOX1*, a peroxisomal β-oxidation enzyme, following simultaneous knockdown of *PEX13* and *PEX14* may suggest a cellular attempt to recover oxidative capacity despite gene knockdown. This aligns with previous studies revealing that high *ACOX1* expression is related to MASLD due to increased peroxisomal β-oxidation demand that accompanies hepatic lipid overload [34].

*SREBF1* expression was markedly increased following *PEX14* knockdown compared with the nonspecific control; however, *FASN* and *SREBF1* were reduced in the *PEX14* knockdown along with the FFA group when compared with the* PEX14* knockdown group [35]. This finding suggests that FFA suppresses compensatory up-regulation due to *PEX14* knockdown. In contrast, no change was observed after *PEX13* knockdown when compared with the nonspecific control; however, after FFA treatment, *SREBF1* was up-regulated in the *PEX13* knockdown compared with the nonspecific control [35, 36,44]. This finding indicates that the up-regulation of SREBF1 is driven by FFA rather than by PEX13 loss itself. Notably, simultaneous knockdowns of *PEX13* and *PEX14* led to increased expression of both *SREBF1* and *FASN* compared with the nonspecific control. This suggests that peroxisomal dysfunction, especially involving the simultaneous disruption of *PEX13* and *PEX14*, strongly enhances lipogenic pathways. These results are aligned with previous studies demonstrating that *SREBF1* and *FASN* are up-regulated in lean MASLD, where dysregulated lipid handling can occur independently of obesity, leading to heightened *de novo* lipogenesis characteristic of the disease [37]. Although our model does not fully represent the clinical phenotype of lean MASLD, the metabolic alterations due to peroxisomal dysfunction resemble mechanisms implicated in contributing to lean MASLD [38].

The reduced expression of *PPARG* following *PEX14* knockdown is especially noteworthy [39]. *PPARG* serves as a key transcriptional regulator of *de novo* lipogenesis and lipid storage, and its reduction may contribute to metabolic dysfunction. This finding is consistent with previous research showing suppressed Pparg expression after Pex14 knockdown in pancreatic β-cell models [7].

*CD36*, a fatty acid transporter, was increased in the *PEX13* knockdown, both with and without FFA treatment, potentially reflecting a compensatory mechanism to preserve lipid metabolic flow [40]. Additionally, *CD36* was significantly elevated following the simultaneous knockdown of *PEX13* and *PEX14*. This data suggests that CD36 induction is primarily contributed by PEX13 depletion, with the contribution of PEX14 being modest only when peroxisomal function is already compromised, which suggests that there is no synergistic interaction. Together, this suggests that *CD36* up-regulation is the cell’s adaptive response to disrupted peroxisomal fatty acid metabolism, leading to increased lipid uptake and possibly lipid accumulation. This observation is consistent with clinical data in MASLD patients, where *CD36* expression is increased, indicating that *PEX13* knockdown may trigger adaptive fatty acid uptake pathways [41].

*PPARA*, a key regulator of lipid catabolism, was significantly up-regulated in the *PEX13* knockdown group with and without FFA treatment, indicating elevated mitochondrial and peroxisomal fatty acid oxidation [42]. *PPARA* also significantly increased after simultaneous knockdown of *PEX13* and *PEX14*, as observed in *CD36*. The combined loss of *PEX13* and *PEX14* amplifies the metabolic stress signal, with *PEX13* loss being the main driver, and *PEX14* contributing only when peroxisomal function is already compromised. The additional increase observed in the double knockdown likely reflects further metabolic stress rather than a true synergistic effect. synergistic interaction between *PEX13* and *PEX14.* This could indicate a cellular effort to mitigate lipid accumulation. By comparison, down-regulated *PPARA* activity has been associated with MASH progression, underscoring the significance of maintaining its expression under lipid overload conditions [43].

Together, these findings indicate that *PEX13* and *PEX14* have overlapping but non-identical roles in maintaining lipid-metabolic homeostasis and that combined loss of both peroxins produces a broader metabolic reprogramming than either knockdown alone. The significant transcriptional response in the simultaneous knockdown reflects the combined metabolic impact of impairing both *PEX13* and *PEX14* peroxisomal import. While the combined knockdown represents greater activation of lipid-metabolism–related genes than either single knockdown, this pattern aligns with the dominant role of *PEX13* and a smaller contribution from *PEX14* when peroxisomal function is already compromised. These suggest the aligned roles of *PEX13* and *PEX14* in maintaining lipid homeostasis, especially under conditions of metabolic stress. These observations contribute to a deeper insight into peroxisomal involvement in MASLD and MASH. However, the current data does not differentiate between additive and synergistic effects.

Additional studies will be required to understand whether *PEX13* and *PEX14* act cooperatively or independently in regulating lipid metabolic pathways. Future studies should investigate the underlying molecular mechanism of these effects, including probable crosstalk between peroxisomes and mitochondria, and analyze whether targeting peroxisomal pathways could offer a positive therapeutic impact on metabolic disorders.

## Supplementary Material

Supplementary Figures S1-S2

## Data Availability

All data generated or analyzed during the present study are included in this article. Further inquiries can be directed to the corresponding authors.

## Open Access

Open access for this article was enabled by the participation of the University of Queensland in an all-inclusive Read & Publish agreement with Portland Press and the Biochemical Society under an agreement with CAUL.

## Abbreviations

ACOX1: acyl-CoA oxidase 1
ACTB: β-actin
CPT1A: carnitine palmitoyltransferase 1A
DNL: *de novo* lipogenesis
FASN: fatty acid synthase
FDB: fluorescence dilution buffer
FFAs: free fatty acids
HPRT: hypoxanthine-guanine phosphoribosyltransferase
IDT: Integrated DNA Technologies
MASH: metabolic dysfunction-associated steatohepatitis
MASLD: metabolic dysfunction-associated steatotic liver disease
MTT: 3-(4,5-dimethylthiazol-2-yl)-2,5-diphenyltetrazolium bromide
PBS: phosphate-buffered saline
PFA: paraformaldehyde
PPARA: peroxisome proliferator-activated receptor alpha
qRT-PCR: quantitative real-time polymerase chain reaction
RNA: ribonucleic acid
RT: room temperature
siRNA: small interfering RNA
SREBF1: sterol regulatory element-binding transcription factor 1
