# Supplementary material for: Loss of peroxisomal membrane proteins PEX13 and PEX14 disrupts fatty acid oxidation and drives lipid imbalance
Source: Biosci Rep. 2026 May 22;46(6):BSR20260048. doi: 10.1042/BSR20260048 (PMC13199795; doi:10.1042/BSR20260048)
Supplement: Supplementary Figures S1-S2 [file BSR-2026-0048_supp.pdf]

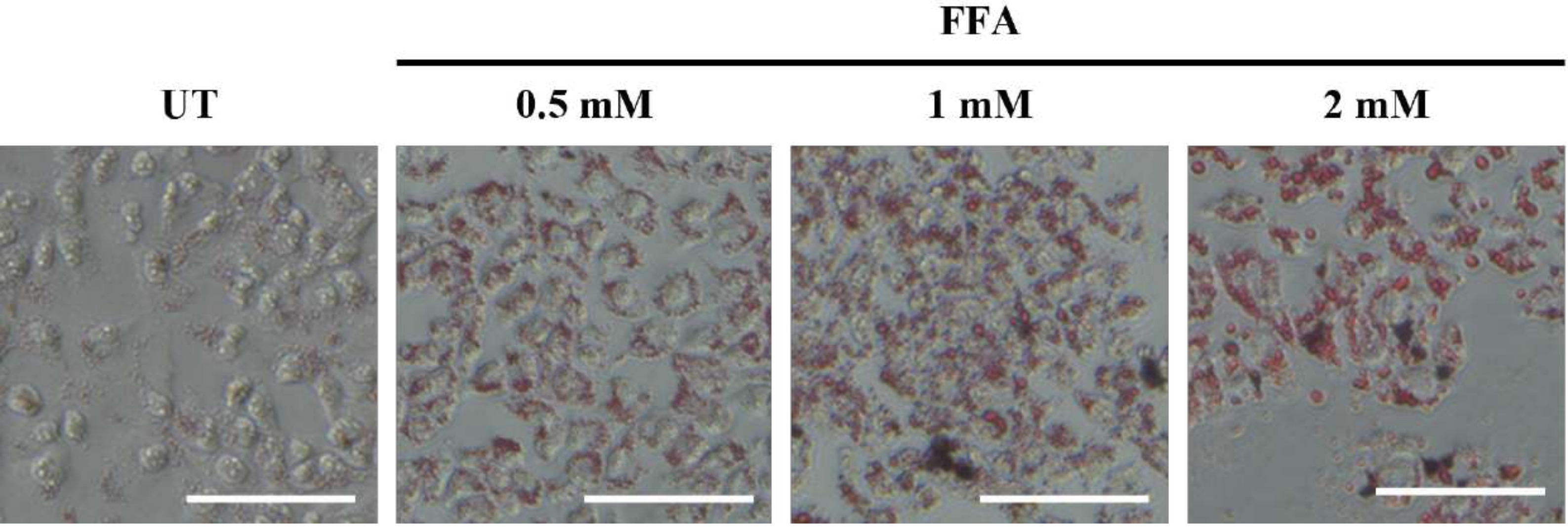
FFA
UT
0.5 mM
1 mM
2 mM

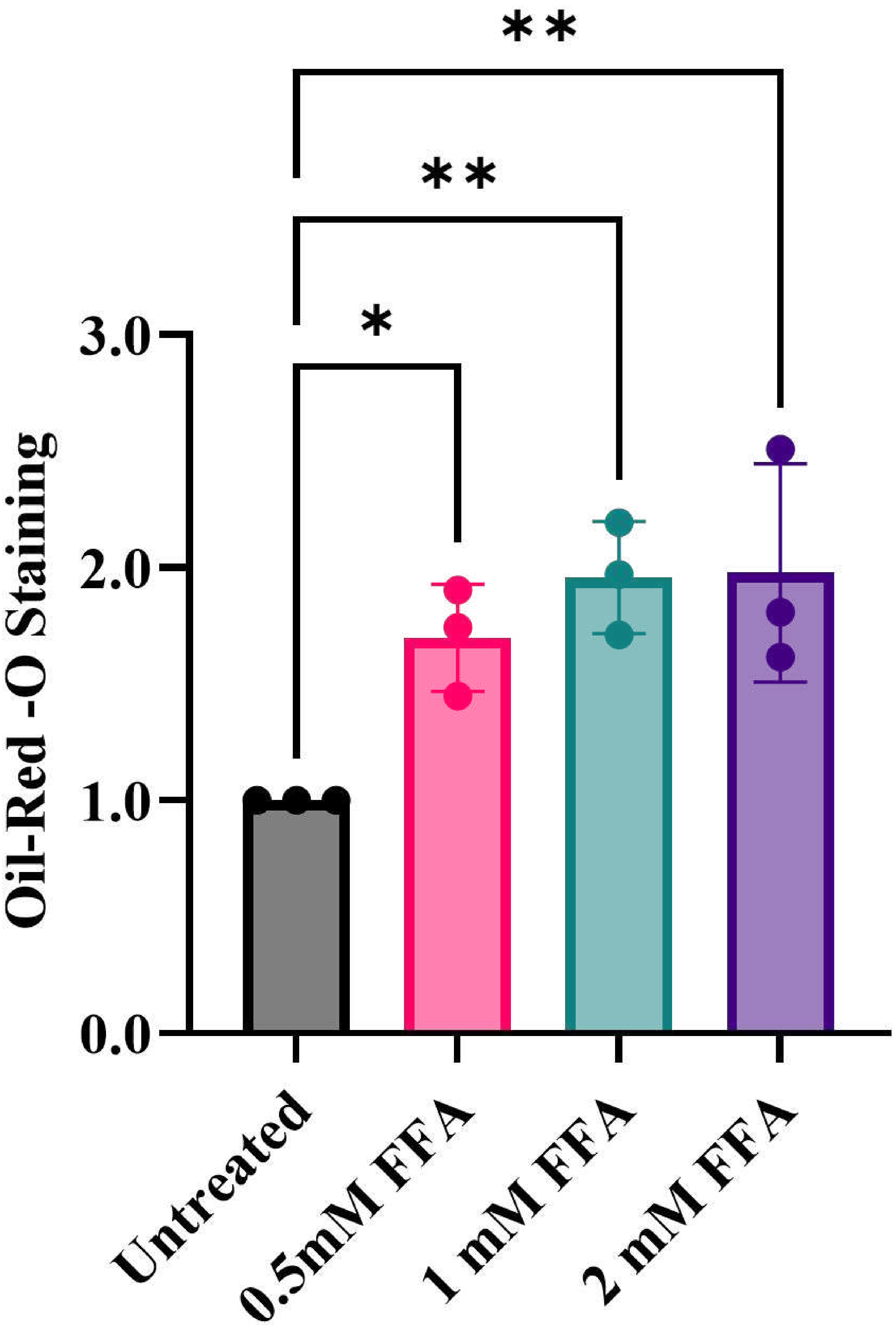

**
**
*
3.0
2.0
1.0
0.0
Oil-Red -O Staining
Untreated
0.5mM FFA
1 mM FFA
2 mM FFA

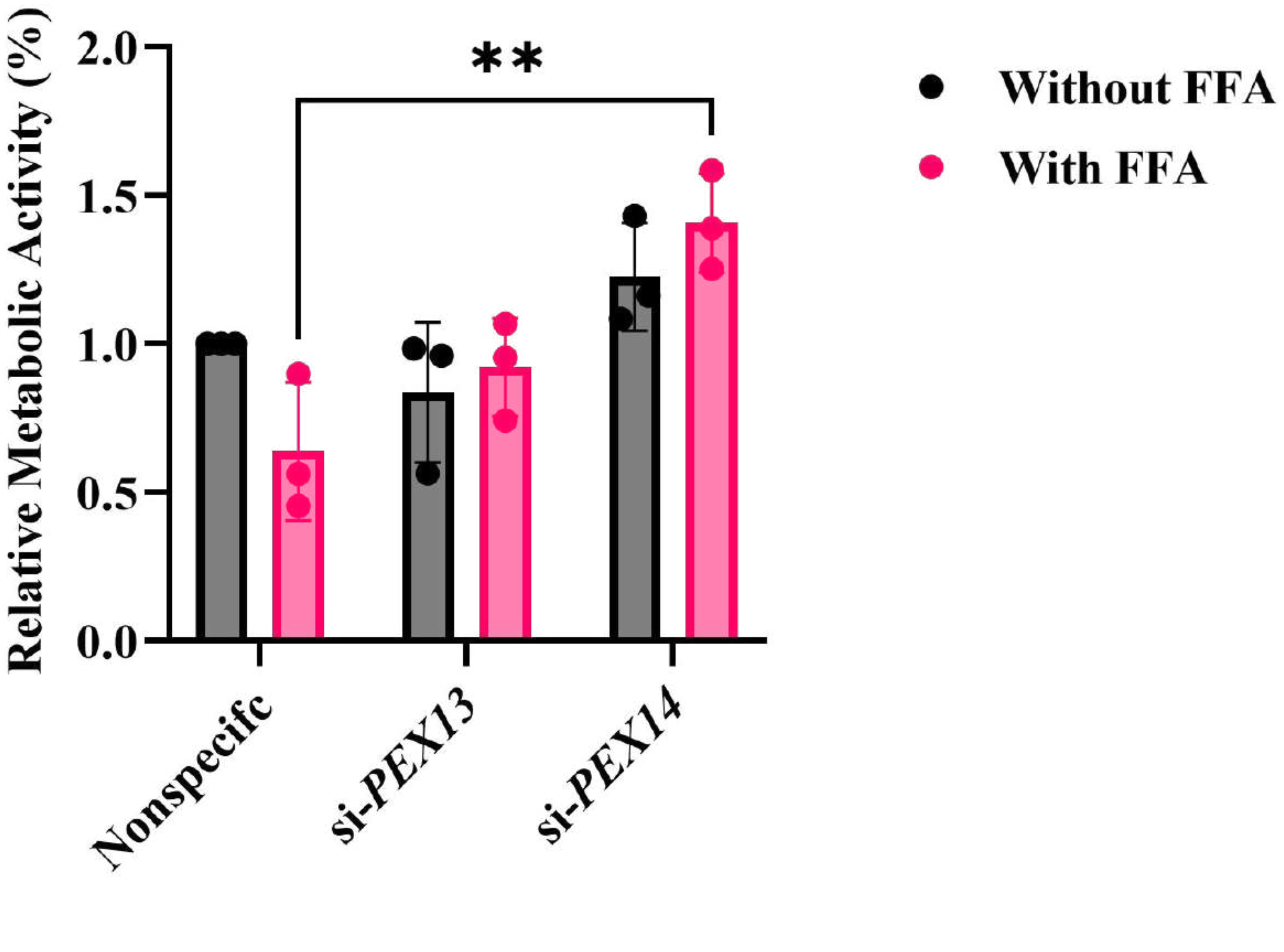

Relative Metabolic Activity (%)
2.0
1.5
1.0
0.5
0.0
**
Nonspecifc
si-PEX13
si-PEX14
Without FFA
With FFA

Supplementary figures

1. *Hepatic steatosis induction*

Figure 1 A: Oil-Red-O staining of neutral triglyceride accumulation and lipid droplets in HUH-7 cells.

HUH-7 cells were treated with 0, 0.5, 1 or 2 mM FFA for 24-hours before being stained with Oil-Red-O and visualized via light microscopy. The neutral triglyceride and lipid droplets increased with increasing FFA concentrations. The images were obtained using a Nikon Eclipse Ts2 inverted microscope. Representative images are shown (n=3). Scale bar = 250 µm.

Figure 1 B: Quantification of neutral triglyceride accumulation and lipid droplets in HUH-7 cells using Oil-Red-O staining.

HUH-7 cells were treated with 0, 0.5, 1 or 2 mM FFA for 24-hours before being stained with Oil-Red-O and visualized via light microscopy. Following light microscopy, the Oil-Red-O stain was extracted from the HUH-7 cells and quantified using a CLARIOstar microplate reader. The relative concentrations of Oil-Red-O were normalized to the untreated control (Untreated), within each biological replicate. The study was performed in technical triplicates with three biological replicate experiments (n=3). One-way ANOVA was conducted comparing all groups with post-hoc Tukey correction. The graph shows the mean and standard error of the mean (SEM). Statistically significant differences are denoted as: * ($p<0.05$) and ** ($p<0.01$).

2. *Impact of siRNA knockdown and FFA treatment on Metabolic Activity*

Figure 2: Quantification of metabolic activity in HUH-7 cells following *PEX* gene knockdown and FFA treatment using MTT assay.

HUH-7 cells were treated with siRNA of peroxisomal genes *PEX13* (si-*PEX13*), *PEX14* (si-*PEX14*), or nonspecific control siRNA (Nonspecific). Cells were then treated with or without 1 mM free fatty acids (FFA) for 24 hours, followed by an MTT assay and visualization using light microscopy. The quantification of the MTT assay was done on CLARIO star Plus Microplate Reader. The relative concentrations of Oil-Red-O were normalised to the nonspecific control (Nonspecific), within each biological replicate. The study was performed in technical triplicate with three biological replicate experiments (n=3). Two-way ANOVA was conducted comparing within or between groups with or without FFA with post-hoc Tukey correction. The graphs show the mean and standard error of the mean (SEM). Statistically significant differences are denoted as: **($p<0.01$).
